# Neoadjuvant treatment of colorectal cancer: comprehensive review

**DOI:** 10.1093/bjsopen/zrae038

**Published:** 2024-05-15

**Authors:** Henry G Smith, Per J Nilsson, Benjamin D Shogan, Deena Harji, Maria Antonietta Gambacorta, Angela Romano, Andreas Brandl, Camilla Qvortrup

**Affiliations:** Abdominalcenter K, Bispebjerg and Frederiksberg Hospital, University of Copenhagen, Copenhagen, Denmark; Department of Molecular Medicine and Surgery, Karolinska Institutet and Dept. of Pelvic Cancer, Karolinska University Hospital, Stockholm, Sweden; Department of Surgery, The University of Chicago Medicine, Chicago, Illinois, USA; Department of Colorectal Surgery, Manchester University NHS Foundation Trust, Manchester, UK; Dipartimento di Diagnostica per Immagini, Fondazione Policlinico Universitario A. Gemelli-IRCCS, Rome, Italy; Dipartimento di Scienze Radiologiche ed Ematologiche, Universita Cattolica del Sacro Cuore, Rome, Italy; Dipartimento di Diagnostica per Immagini, Fondazione Policlinico Universitario A. Gemelli-IRCCS, Rome, Italy; Department of General, Visceral and Transplantation Surgery, University Hospital Heidelberg, Heidelberg, Germany; Department of Oncology, Rigshospital, University of Copenhagen, Copenhagen, Denmark

## Abstract

**Background:**

Neoadjuvant therapy has an established role in the treatment of patients with colorectal cancer. However, its role continues to evolve due to both advances in the available treatment modalities, and refinements in the indications for neoadjuvant treatment and subsequent surgery.

**Methods:**

A narrative review of the most recent relevant literature was conducted.

**Results:**

Short-course radiotherapy and long-course chemoradiotherapy have an established role in improving local but not systemic disease control in patients with rectal cancer. Total neoadjuvant therapy offers advantages over short-course radiotherapy and long-course chemoradiotherapy, not only in terms of increased local response but also in reducing the risk of systemic relapses. Non-operative management is increasingly preferred to surgery in patients with rectal cancer and clinical complete responses but is still associated with some negative impacts on functional outcomes. Neoadjuvant chemotherapy may be of some benefit in patients with locally advanced colon cancer with proficient mismatch repair, although patient selection is a major challenge. Neoadjuvant immunotherapy in patients with deficient mismatch repair cancers in the colon or rectum is altering the treatment paradigm for these patients.

**Conclusion:**

Neoadjuvant treatments for patients with colon or rectal cancers continue to evolve, increasing the complexity of decision-making for patients and clinicians alike. This review describes the current guidance and most recent developments.

## Teaser

Neoadjuvant treatment for colon and rectal cancer continues to evolve. This comprehensive review summarizes the current guidance for neoadjuvant treatment and the promising avenues for future developments.

## Introduction

Neoadjuvant treatment refers to cancer therapies that are given prior to a potentially curative definitive local treatment. The treatment modalities used, and the indications for using them, differ according to a number of factors, including tumour location, tumour biology and the intended definitive treatment. The influence of these different factors is well illustrated in patients with colorectal cancer. Broad distinctions can be made between groups of these patients, with neoadjuvant therapy having a more established role in patients with rectal than colon cancer. However, a greater appreciation of the heterogeneity of tumour biology and the emergence of novel therapeutic options demands a more nuanced approach. The challenges are identifying which patients will benefit most from which treatment and, in those patients with good responses, what type of definitive local treatment, if any, is required. This narrative review aims to summarize the current evidence and guidance for neoadjuvant treatment in patients with colon or rectal cancers, describe the recent advances in this field and discuss the most promising avenues for future developments.

## Rectal cancer

### The evolution of the current treatment for rectal cancer

The current management of rectal cancer is a product of refinements in both surgical technique and its combination with chemo- and/or radiotherapy that have occurred during the past 40 years. The aim of these refinements was primarily to reduce the risks of local recurrence following surgery. Prior to the 1980s, local recurrence was a common event, with reported rates of between 30% and 40%^1,2^. Patients with local recurrence often had intractable pain and severe urogenital dysfunction, symptoms that were difficult to ameliorate. Given the difficulty in treating established local recurrences, significant efforts were made to identify strategies that could improve local disease control.

Total mesorectal excision (TME) is arguably one of the most significant of these refinements and to this day still represents one of the most important technical advances in cancer surgery. Previously, the standard approach for surgical resection of rectal cancers had involved blunt, and often blind, dissection (or manual extraction) of the mesorectum, the fibrofatty tissue surrounding the rectum that contains its blood vessels and draining lymph nodes. Consequently, variable amounts of the mesorectum were left *in situ*, which Bill Heald and his collaborators in Basingstoke hypothesized as the source of local recurrence^3^. The aim of TME was to remove the entire mesorectum within its fascial envelope by using sharp dissection of embryological planes under direct vision. This dramatically reduced local recurrence from rates of up to 40% to the initial TME series in which 115 patients treated with curative intent reported a 5-year local recurrence rate of just 3.7%^4^. A later larger series from Basingstoke of over 500 patients not only confirmed these low rates of recurrence but also demonstrated an associated improvement in overall survival, which at 5 years following TME was 68% for all-comers compared with a national figure of 38%^5^. In the light of these results, TME was increasingly adopted as the standard approach for surgical resection of rectal cancers and viewed by many as the benchmark against which neoadjuvant treatments should be measured.

However, before the widespread adoption of TME, other significant trials were performed investigating the potential benefits of radiotherapy. A randomized trial from Uppsala, Sweden demonstrated the superiority of preoperative compared to postoperative radiotherapy^6^. Despite the lower total radiation dose, preoperative irradiation with 25 Gy was associated with a significant reduction in 5-year local recurrence rate when compared with postoperative irradiation with 60 Gy (14.3% *versus* 26.8%, *P* = 0.021). The Swedish Rectal Cancer trial followed and confirmed that preoperative radiotherapy led to reduced risks of local recurrence at 5 years when compared with surgery alone (11% *versus* 27%, *P* < 0.001)^7^. However, a shared criticism of these trials was that they were performed in patients who had undergone what was now generally accepted as suboptimal surgery. As such, it was unclear what benefit radiotherapy would have now that surgical techniques had been refined. The Dutch TME trial, where surgical resection was standardized and quality controlled, aimed to answer this question^8^. Even though the overall local recurrence rate was lower than in the previous trials, a significant benefit was still seen with preoperative radiotherapy, which even at 10 years follow-up was associated with a local recurrence rate of just 2.4%, compared with 8.2% with TME alone (*P* < 0.001)^9^.

Although these trials established the benefit of preoperative radiotherapy in reducing local recurrence, with the exception of The Swedish Rectal Cancer Trial, no corresponding improvements in overall survival had been shown. This is perhaps not unexpected, given that radiotherapy is a local rather than systemic treatment. The European Organization for Research and Treatment of Cancer (EORTC) 22921 trial explored the potential benefit of combining radiotherapy and chemotherapy, using a 2 × 2 factorial design where patients were randomized in four groups to preoperative radiotherapy or preoperative chemoradiotherapy, with or without postoperative chemotherapy^10^. Disappointingly, no differences in terms of systemic relapse or overall survival were noted between the interventional arms in this study. However, patients who received chemotherapy, either before or after surgery, had lower local recurrence rates when compared to those treated with preoperative radiotherapy alone, a difference that was maintained at 10-year follow-up^11^. At the same time, The German Rectal Cancer Trial investigated whether chemoradiotherapy was best given preoperatively or following surgery^12^. This trial demonstrated that preoperative chemoradiotherapy was not only better tolerated but also associated with reduced rates of local recurrence (5-year local recurrence rate of 6% *versus* 13%, *P* = 0.006). However, no difference in overall survival was seen. The MRC CR07 followed shortly after and compared preoperative radiotherapy to selective use of postoperative chemoradiotherapy, given only to patients with involvement of the circumferential resection margin (≤1 mm)^13^. Although preoperative radiotherapy was associated with a lower recurrence rate (3-year local recurrence rate of 4.4% *versus* 10.6%, *P* < 0.001), once again no difference in overall survival was noted between the groups.

A further critical advance was the refinement of clinical staging, allowing patients to be stratified according to their risks of relapse in order to identify those who would benefit most from neoadjuvant treatment. Preliminary studies in the late 1990s and early 2000s demonstrated the value of MRI in accurately determining T and N stage in rectal cancers, as well as visualizing involvement of the mesorectal fascia (MRF) and identifying other risk factors, such as extramural venous invasion (EMVI)^14,15^. These studies were followed by the MERCURY trial, which found that MRI could reproducibly predict which patients would have clear or involved circumferential resection margins following TME^16^. With longer-term follow-up, it became evident that MRI-based assessments of the circumferential resection margin outperformed TNM-based staging in predicting both local and distant recurrence, confirming the value of MRI in stratifying patients to neoadjuvant treatment based on their risk profile^17,18^.

The lessons learnt from these landmark studies provide several themes that underpin the modern-day neoadjuvant treatment for rectal cancer. Neoadjuvant treatments are a supplement to and not a substitute for optimal surgical technique. Preoperative therapies are more effective than postoperative therapies, which are less well tolerated. Neoadjuvant treatments improve local but not distant disease control. Although some of these themes remain constant, recent developments have begun to challenge others, as will be discussed later.

### What are the current indications for neoadjuvant therapy?

As described above, evidence has shown that neoadjuvant therapy reduces local recurrence rates following surgery, but corresponding improvements in overall survival have been elusive^6–10,12,13^. As such, neoadjuvant treatment should primarily be viewed as a means to reduce surgical complexity and to improve locoregional disease control. The aim of such treatment can be to sterilize visible or occult extra-mesorectal pelvic spread, induce downstaging/downsizing in order to improve R0 resection rates but also, potentially, to increase possibilities for sphincter preservation.

However, given the impressive rates of local disease control following TME, it is clear that neoadjuvant therapy is not needed in all patients with rectal cancer^5^. Furthermore, neoadjuvant therapy is not without consequence, being associated with increased rates of bowel and sexual dysfunction, with associated negative impacts on quality of life^19,20^. Several adverse features can be used to identify patients at higher risk of recurrence, who may stand to benefit most from neoadjuvant therapy. Such features include tumour height, cT-stage, relation of the tumour to the MRF, cN-stage, presence or absence of EMVI, and if any enlarged suspicious lateral lymph nodes are present^18,21,22^. These features should be discussed at the pretherapeutic multidisciplinary tumour board and a prerequisite to that discussion is meticulous staging including high-quality MRI^23^. For cT3 stage tumours, subgrouping according to the extent of invasion beyond the muscularis propria seen on MRI into those ≤5 mm (cT3a/b) and those >5 mm (cT3c/d) is desirable^24^.

Some variation is noted in the current indications for neoadjuvant treatment between international guidelines^25–27^. A more intensive approach is taken in North America, with neoadjuvant therapy also recommended for patients with cT1–2 tumours if there is clinical suspicion of nodal involvement^27^. Critics of this strategy state that it may lead to overtreatment of patients with lower-risk cancers and question the relative value of cN stage compared to other adverse features. The MERCURY trial demonstrated that local recurrence rates of <2% could be achieved with TME alone in patients with cT3 cancers regardless of nodal stage if other adverse features were absent (≤5 mm extramural spread, absent EMVI)^18^. The current European guidelines are more restrictive, with neoadjuvant treatment reserved for patients with cT3c/d to cT4 tumours, those with a threatened MRF, lateral lymph node involvement or EMVI^26^.

Despite these differences, most clinicians involved in the care and management of rectal cancer patients would probably agree that patients with a ‘locally advanced’ tumour should receive, or at least be considered for, neoadjuvant therapy. In patients with cT4 cancers, evidence of MRF and/or lateral lymph node involvement, the indication for neoadjuvant therapy is strong and commonly accepted. There is likely consensus that there is a wider indication for neoadjuvant therapy for distal tumours, meaning that also cT3a/b or even cT2 could receive treatment when distal. EMVI has been shown to be an independent poor prognostic factor and, according to the current European guidelines, its presence should be considered a specific indication for neoadjuvant therapy^26,28^.

### Which treatment modality should be used?

Until recently, neoadjuvant therapy for rectal cancer consisted of two main strategies—short-course radiotherapy (SCRT) with 25 Gy given in five fractions or long-course chemoradiotherapy (CRT) with 45–50 Gy given in 25–28 fractions with concurrent low-dose fluoropyrimidine-based chemotherapy, which functions as a radiosensitizer. Although there are robust data to support both regimens, few studies have investigated whether one is superior to the other. In The Polish Rectal Cancer trial, 312 patients with resectable cT3/4 rectal cancers were randomized to SCRT or CRT^29^. The primary outcome of the study was sphincter preservation, which did not differ between the groups. Additionally, no statistically significant difference in local recurrence rates was noted (4-year local recurrence rate of 10.6% for SCRT *versus* 15.6% for CRT, *P* = 0.210). The Trans-Tasman Radiation Oncology Group trial randomized 326 patients with cT3 rectal cancers to SCRT or CRT, where the primary outcome was local recurrence rate at 3-year follow-up^30^. Once again, no statistically significant difference in local recurrence rates was noted between regimens (7.5% for SCRT *versus* 4.4% for CRT, *P* = 0.240). Neither of these trials demonstrated any differences in systemic relapse rates or overall survival^29,30^. These data suggest that SCRT and CRT are comparable in terms of reducing the risks of local recurrence. However, there is some evidence to suggest that the addition of chemotherapy may be of greater benefit in patients with cancers that threaten the MRF, where downstaging prior to surgery is desired. In a study of 207 patients with either non-resectable primary cT4 or locally recurrent rectal cancers who were randomized to preoperative radiotherapy at a dose of 50 Gy with or without concomitant chemotherapy, CRT was associated with a higher R0 rate (84% *versus* 64%, *P* = 0.009), with associated improvements in 5-year local control rates (82% *versus* 67%, *P* = 0.030) and cancer-specific survival (72% *versus* 55%, *P* = 0.020) when compared to radiotherapy alone^31^.

Late toxicities and impaired quality of life can occur after both CRT and SCRT, but no head-to-head comparative study of these outcomes is available^19,20^. In addition, a caveat to the findings above is that many of the published data are based on radiation techniques and volumes, which are less commonly used in clinical practice today. The use of modern radiotherapy techniques, such as intensity-modulated radiotherapy (IMRT) should be encouraged, and novel developments such as proton radiotherapy may also play a role in the future^32^.

### Total neoadjuvant therapy

A further development in recent years is the emergence of total neoadjuvant therapy (TNT) as an alternative to SCRT or CRT. Despite a lack of convincing evidence, adjuvant chemotherapy following rectal cancer surgery has been used in many countries with the aim of reducing the risk of systemic recurrence^33^. However, poor compliance with postoperative chemotherapy has been a persistent issue^10,12^. TNT aims to address this limitation by delivering full-dose chemotherapy preoperatively, either in the context of SCRT or CRT, where it may be given before or after irradiation, as induction or consolidation chemotherapy respectively^34^. In doing so, the hope was that compliance with full-dose chemotherapy could be improved, leading to reduced risks of systemic recurrence and, ultimately, improved survival.

**Fig. 1:**
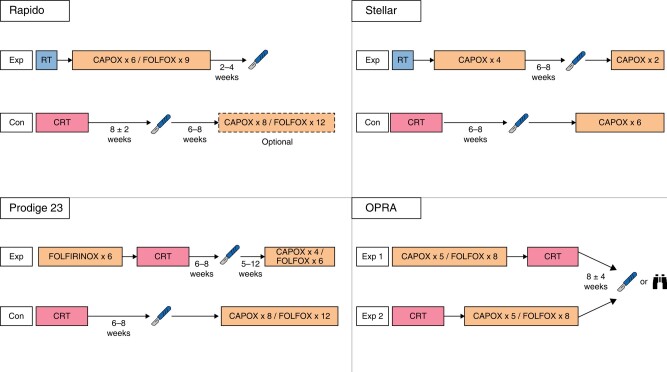
The total neoadjuvant therapy regimens used in the RAPIDO, PRODIGE 23, STELLAR and OPRA trials Exp, experimental arm; Con, control arm; RT, short-course radiotherapy; CRT, long-course chemoradiotherapy. Patients with clinical complete or near-complete responses in the OPRA trial were offered non-operative management (NOM) as an alternative to surgery. NOM was also offered to patients with clinical complete response in the STELLAR trial.

The results of recent trials of TNT have been encouraging and a growing enthusiasm for adopting TNT as the standard-of-care for rectal cancer can be perceived. The different regimens used in these trials are summarized in *Fig. 1* and their results summarized in *Table 1*. The PRODIGE23 trial randomized 461 patients, stage cT3–4 N0–2 M0, to induction chemotherapy with six cycles of FOLFIRINOX followed by CRT or CRT alone^35^. Postoperative adjuvant chemotherapy was prescribed to both groups regardless of histopathological findings. Interestingly, only 18% and 16% cT4 tumours were included in the two groups respectively and about a quarter of patients had cN2 staging. With a median follow-up of 46.5 months a statistically significantly improved disease-free survival was reported for TNT (76% *versus* 69%, *P* = 0.034). This was mostly due to a reduction in the rate of distant metastases (17% for TNT *versus* 25% for CRT). However, no corresponding differences in 3-year overall or cancer-specific survival were noted. Pathological complete response (pCR) rates were also increased (28% for TNT *versus* 12% for CRT, *P* < 0.001). Despite this, and perhaps due to the high R0 resection rates observed in both arms, no difference in local recurrence rates was reported, at 6% and 4% (*P* = 0.560) respectively.

**Table 1:** A summary of trials of total neoadjuvant therapy in patients with rectal cancer

Study (year)	Inclusion criteria	*N*	Primary endpoint	Treatment arms	pCR rate (%)	R0 rate (%)	DFS (%)	OS (%)	DM (%)	LRR (%)
RAPIDO (2021)	At least one of: cT4a/b; EMVI; cN2; MRF+; LLN	920	3-year DRTF	SCRT → CT→TME	28	90	23.7	89.1	20.0	8.3
				CRT → TME	14	90	30.4	88.8	26.8	6.0
PRODIGE 23 (2021)	cT3/4	461	3-year DFS	CT → CRT→TME	28	95	76	91	17	4
				CRT→TME	12	94	69	88	25	6
STELLAR (2022)	cT3/4 or cN+	599	DFS	SCRT → CT→TME	17.2	91.5	64.5	86.5	22.8	8.4
				CRT → TME	13.9	87.8	62.3	75.1	24.7	11.0
OPRA (2022)	cT3/4 or cN+	324	DFS	CT → CRT→TME	NS	91	76	NS	16	6
				CRT → CT→TME	NS	88	76	NS	18	6

All oncological outcomes are reported at 3-year follow-up. CRT, long-course chemoradiotherapy; CT, chemotherapy; DFS, disease-free survival; DM, distant metastases; DRTF, disease-related treatment failure; EMVI, extramural venous invasion; LLN, lateral lymph node involvement; LRR, locoregional recurrence; MRF, mesorectal fascia; NS, not specified; OS, overall survival; pCR, pathological complete response; SCRT, short-course radiotherapy; TME, total mesorectal excision.

The STELLAR trial, contrary to PRODIGE23, included SCRT in the TNT schedule^36^. Almost 600 patients with stage cT3–4 and/or N + M0 were randomized to either CRT or TNT with SCRT followed by four cycles of capecitabine and oxaliplatin (CAPOX) as consolidation chemotherapy. The cT4 rate among included patients was even lower than PRODIGE23, whereas the cN2 rate was somewhat higher. No statistically significant differences in disease-free survival or in the rates of distant metastases or local recurrence were reported (at a median follow-up of 35 months), but an increased complete response rate (either pathological or clinical) was again observed in the TNT arm (21.8% *versus* 12.3%, *P* = 0.002).

The larger RAPIDO trial is characterized by the inclusion of more advanced tumours in comparison with PRODIGE23 and STELLAR^37^. In RAPIDO, patients in whom MRI described at least one of cT4, cN2, threatened MRF, EMVI, or lateral lymph nodes suspicious of involvement were randomized to either CRT or TNT with SCRT followed by six cycles of CAPOX as consolidation chemotherapy. Postoperative chemotherapy was only prescribed for CRT patients, and only in some centres. Of the patients included in this study, 32% had cT4 cancers, 62% had a threatened MRF and 65% were cN2. With a median follow-up of 4.6 years, disease-related treatment failure was significantly reduced in the TNT arm. This was primarily driven by a statistically significant reduction in distant metastases (20% *versus* 27%, *P* = 0.005). With regard to pCR, TNT yielded a rate of 28% compared to 14% after CRT (*P* < 0.001). A difference in local recurrence rate in favour of CRT was initially non-significant, but with a longer follow-up, local recurrence after R0/R1 resection was 10% in TNT and 6% after CRT (*P* = 0.027)^38^. Despite this, the initial reductions in the rate of distant metastases were still maintained (23.0% *versus* 30.4% at 5 years, *P* = 0.011).

These three trials all represent valuable contributions to the understanding of TNT and its use. However, direct comparison between them is difficult because of the differences in treatment schedules and study populations. TNT appears to offer additional benefits to SCRT or CRT both in terms of the rate of pCR and in reducing the risks of systemic relapse, but questions remain as to which regimen is best. Given that the RAPIDO trial included a greater proportion of more advanced tumours but still achieved comparable results to the PRODIGE23 trial, one could argue in favour of a TNT regime based on consolidation rather than induction chemotherapy. However, the poorer local control rates seen with longer follow-up of the RAPIDO trial are a cause for concern. The reasons for this are unclear, especially given the higher rates of pCR in the interventional arm. Breaches of the mesorectal plane were more common in patients receiving TNT (22% *versus* 15%), which may suggest that treatment made the mesorectum more friable. However, the higher rate of breaches was not associated with an increase in R1 resections. Fragmentation has been suggested as an alternative explanation, where a heterogenic response to therapy in large tumours results in small islands of viable cancer cells within a tumour that macroscopically appears to have responded to therapy^39^. As such, questions remain as to which TNT regimen, if either, should be preferred.

A further point of contention lies in which patients should be given TNT. The ESMO guideline published in 2017 recommends TNT only for patients with cT3 cancers with MRF+, cT4 cancers, and those with positive lateral lymph nodes, whereas the more recent National Comprehensive Cancer Network (NCCN) guideline suggests TNT also as an option for less advanced tumours^26,27^. Although TNT will undoubtedly play a major role in the management of patients with rectal cancer in the coming years, the optimal indications for the different regimens with respect to surgical complexity, oncological risk and long-term functional outcomes are still to be determined.

### Neoadjuvant chemotherapy

In contrast to TNT, several recent studies have investigated a de-escalation of neoadjuvant therapy, whereby chemotherapy is used as a single agent modality. The results of these trials are summarized in *Table 2*. Proponents for this strategy argue that the low rates of local recurrence seen after TME have reduced the potential benefit of radiotherapy in terms of local disease control. However, the potential cost of radiotherapy, in terms of both short- and long-term toxicities, remains^19,20,40^. These toxicities could potentially be avoided in patients whose cancers do not require downstaging prior to TME, where systemic chemotherapy could still be given to reduce the risks of systemic relapse.

**Table 2:** A summary of trials of neoadjuvant chemotherapy in patients with rectal cancer

Study (year)	Inclusion criteria	*N*	Primary endpoint	Treatment arms	pCR rate (%)	R0 rate (%)	DFS (%)	OS (%)	LRR (%)
PROSPECT (2023)*	cT2N+ or cT3	1194	DFS	FOLFOX→TME	21.9	90.4	80.8	89.5	1.8
				CRT→TME	24.3	91.2	78.6	90.2	1.6
FOWARC (2019)†	cT3/4 or cN+	495	DFS	CRT →TME	14.0	90.7	72.9	91.3	8.0
				CRT (mFOLFOX)→TME	27.5	89.9	77.2	89.1	7.0
				mFOLFOX→TME	6.5	89.4	73.5	90.7	8.3
CONVERT (2023)	cT2N+ or cT3/4	663	LRFFS	CAPOX→TME	11.0	99.6	NS	NS	NS
				CRT → TME	13.8	99.6	NS	NS	NS

*Oncological outcomes reported at 5-year follow-up. †Oncological outcomes reported at 3-year follow-up. CRT, long-course chemoradiotherapy; DFS, disease-free survival; LRR, locoregional recurrence; LRFFS, locoregional failure-free survival; NS, not specified; OS, overall survival; pCR, pathological complete response; TME, total mesorectal excision.

The PROSPECT trial is the most recently published study on this topic, in which 1194 patients were randomized to either neoadjuvant chemotherapy with fluorouracil, leucovorin and oxaliplatin (FOLFOX) or CRT^41^. Patients in the FOLFOX arm were restaged prior to surgery, with CRT given to those whose tumours had decreased by less than 20% or those who could not complete ≥5 cycles. Adjuvant chemotherapy following surgery was suggested for both arms but was not mandatory. The included patients represented an intermediate risk group, with mid or upper rectal cancers of cT2N1–2 or cT3N0–2 staging. The primary endpoint was the non-inferiority of neoadjuvant FOLFOX in terms of disease-free survival. Most patients had cT3 node positive cancers (52.8%), and a minority (just over 15%) had low rectal cancers. A total of 53 patients (9.1%) in the FOLFOX arm also received neoadjuvant CRT, most commonly due to poor response at restaging. Most patients also received adjuvant chemotherapy, with no differences noted between the two groups (74.9% in the FOLFOX arm, 77.9% in the CRT arm). The primary endpoint was met, with a 5-year disease-free survival of 80.8% in the FOLFOX arm compared with 78.6% in the CRT arm. Similarly, no differences in overall survival or local recurrence rates were noted between these groups. The pCR rate was slightly higher in the CRT arm (24.3% *versus* 21.9%), although no differences were noted in the rates of R0 resection. Importantly, patient-reported outcomes were also collected from both study arms, with patients in the FOLFOX arm reporting lower incidences of various adverse effects, such as diarrhoea, nausea, vomiting and fatigue^42^.

The findings are similar to those of the FOWARC trial, which was actually designed to investigate whether the use of neoadjuvant FOLFOX alone or in combination with radiotherapy was superior to CRT in terms of 3-year disease-free survival^43^. The inclusion criteria for this trial allowed for more advanced cancers. Of the 495 patients included, one in three patients had cT4 cancers, one in four patients had cN2 disease and more than half had low rectal cancers. A further difference was that adjuvant chemotherapy was included as standard in the trial protocol. Although patients treated with FOLFOX alone had the lowest rates of pCR (6.6% compared to 14.0% with CRT), no corresponding differences in R0 resection rates were noted. With 3-year follow-up, no differences in local recurrence rates, disease-free survival or overall survival were noted between the interventional groups^44^. Although the trial's primary endpoint was not met in that the FOLFOX regimes were not found to be superior to CRT, outcomes were similar in patients treated with either FOLFOX or CRT both in terms of local recurrence (8.0% *versus* 8.3%) and overall survival (91.3% *versus* 90.7%).

In contrast to these studies, the CONVERT trial investigated the non-inferiority of neoadjuvant CAPOX compared to CRT. The preliminary results of this study have been reported but do not include the primary endpoint, which was 3-year local–regional failure-free survival^45^. As in the FOWARC trial, this study included more advanced cancers and included adjuvant chemotherapy as standard in both arms. Of the 589 patients who received treatment, one in four had cT4 cancers and/or cN2 disease and just over 40% had low rectal cancers. Although there was no significant difference in pCR rate between CAPOX and CRT (11.0% *versus* 13.8%), the proportion of patients achieving tumour regression grade 0–1 was higher after CRT (23.2% *versus* 36.8%, *P* < 0.001). However, once again, no differences in R0 rates were noted between the groups.

Although these three studies provide some evidence to suggest that neoadjuvant chemotherapy is non-inferior to CRT in selected patients with rectal cancer, whether they represent a true de-escalation of therapy depends on the context in which these studies are interpreted. The PROSPECT study was conducted in North America, where current guidelines recommend the consideration of TNT for patients who met the study's eligibility criteria^27^. As such, the use of neoadjuvant chemotherapy would represent a considerable de-escalation of therapy, which may be expected to reduce treatment-related toxicities without compromising oncological outcomes. However, as stated above, a less-intensive approach is taken in Europe, where many of these patients would be treated with TME alone^26^. In this setting, these trials actually represent an escalation of current therapy. This may be of questionable oncological benefit, given that the MERCURY study suggests that, in a similar patient group to that included in PROSPECT, similar local recurrence-free and disease-free survival can be achieved with TME alone^17^.

### Is formal surgical resection still needed?

Alongside these developments in neoadjuvant regimens, there has been increasing interest in organ preservation, whereby ‘watch and wait’ surveillance, also referred to as non-operative management (NOM), is preferred to surgical resection. This strategy may be adopted following a clinical complete response (cCR) after neoadjuvant therapy, offering a personalized approach to a selected group of patients with rectal cancer. One of the key drivers in the development of this strategy was the outcomes of patients undergoing TME after CRT with complete or near-complete pathological responses. These patients not only had significantly better long-term outcomes than ‘non-responders’ but also had low rates (<5%) of positive lymph nodes in the mesorectum^46,47^. As such, many of these patients could be considered cured by CRT, where surgical resection may not offer much, if any, additional oncological benefit. NOM not only has the potential to spare patients from unnecessary surgery but also to improve their quality of life, as it is associated with fewer risks of morbidity, mortality and functional impairment^48–51^.

The first report of the long-term outcomes of NOM was published in 2004, where a total of 71 patients who developed a cCR after CRT were enrolled in an intensive surveillance programme rather than surgery^48^. At a median follow-up of almost 5 years, only two patients (2.8%) developed local tumour regrowth and only three patients (4.2%) developed distant metastases. Most importantly, however, was that no differences in recurrence rates or mortality rates were observed between these patients who underwent NOM and those patients found to have pCR after CRT and TME. Several other cohort studies have since been published, all of which reported the outcomes of NOM after neoadjuvant treatment with CRT. Meta-analyses of these studies have demonstrated no difference in the rates of non-regrowth recurrence or cancer-specific mortality between patients undergoing NOM after cCR and those found to have pCR after TME^52,53^. Although there are limited data regarding NOM after cCR following SCRT, the available studies report similar outcomes, both in terms of local tumour regrowth and overall survival^54,55^.

Given its association with improved rates of pCR, there has been much interest in using TNT as the basis for NOM strategies. This potential was investigated in the recently published OPRA study, which compared two different TNT schedules in 324 patients with stage II or III rectal cancer^56^. Patients were randomly assigned to either an induction (chemotherapy followed by CRT) or consolidation regime (CRT followed by chemotherapy). After restaging, those with a cCR (or near complete response) were assigned to NOM, whereas the remaining patients underwent TME. The primary endpoint of the study was disease-free survival. Organ preservation, defined as TME-free survival, was included as a secondary endpoint. No difference was found in disease-free survival between the two TNT regimes, which at 3 years was 76% in both arms. The rate of NOM was also similar at 71% for the induction regime and 76% for the consolidation regime. However, despite these similarities, the rates of tumour regrowth were lower in the consolidation TNT group (27% *versus* 40%), which translated into a significantly better rate of organ preservation at 3-year follow-up (53% *versus* 41%, *P* = 0.01). A further significant finding was that no differences were seen in disease-free survival both at 3-year and 5-year follow-up between patients undergoing TME after initial restaging and those undergoing TME after tumour regrowth^56,57^. Taken together, the OPRA trial suggests that NOM is oncologically safe in patients with cCR or near-complete response after TNT and that this strategy can achieve long-term organ preservation in more than half of these patients.

An alternative organ-preserving strategy is the combination of neoadjuvant therapy with a local excision (LE) of the primary tumour rather than TME. This approach was first assessed in the GRECCAR 2 trial, where patients with cT2–3 rectal cancers with a good clinical response to CRT (defined as ≤2 cm residual tumour) were randomized to LE or TME^58^. A total of 145 patients were included in the final analyses, of whom 74 were allocated to initial LE after CRT. However, 26 (35%) of these patients underwent completion TME, either due to poor response within the primary tumour (ypT2–3) or an R1 margin after LE. No difference was noted in oncological outcomes between the trial arms, although these analyses were complicated by the number of patients crossing over between the interventional arms. More recently, the GRECCAR group have published a prospective observational series of patients with cCR or near-complete response who underwent LE after CRT^59^. Of the 257 patients who underwent LE, a theoretical indication for completion TME was found in 104 (42%), although this was only actually performed in 42 patients (16.3%). Interestingly, whereas patients undergoing completion TME had higher rates of systemic relapse, no difference in local control rates was noted between the groups. The role of LE in this setting has also been investigated by the TREC study, where the primary endpoint was the feasibility of randomizing patients to TME or organ preservation with SCRT followed by LE^60^. A total of 55 patients were randomized, with 27 allocated to the organ-preservation arm. Organ preservation was achieved in 70% of these patients, with TME performed in the remainder either due to adverse features in the LE specimen (*n* = 5) or because LE was not technically feasible (*n* = 3). Local recurrence occurred in three patients allocated to organ preservation, which was salvageable in two patients. Although the study was not powered to detect any differences in oncological outcomes, patients allocated to organ preservation reported better functional outcomes when compared to those undergoing TME. The STAR-TREC trial aims to build on these results, with patients who prefer an organ-preserving approach being randomized to SCRT or CRT, which is followed by NOM in patients with cCR or LE in patients with a partial response^61^.

### Local tumour regrowth after NOM

As highlighted above, one potential issue with an NOM strategy is local tumour regrowth (LTR). This is defined as clinically recognizable disease following the achievement of a previous cCR. LTR includes the presence of progressive disease within the rectal wall, the mesorectal compartment or the lateral internal iliac/obturator lymph nodes^62^. The incidence of LTR is estimated to be 25–30%, with the risk of regrowth being the highest in the first 2–3 years after cCR^51,52^. Extrarectal LTR is uncommon, with an incidence of 3%^51^. This can present as nodal disease, extranodal tumour deposits or EMVI. The main risk factor for LTR within the first 12 months is advanced T stage at the index presentation; following this the impact of T stage of LTR diminishes^63,64^. Diagnosis of LTR consists of clinical examination, endoscopic evaluation and radiological assessment and, more rarely, an elevated carcinoembryonic antigen (CEA). Early signs of LTR can include superficial ulceration at endoscopy and scar thickening, depth angle increase and areas of mixed intensity on MRI imaging^65^.

Approximately 85% of LTR are salvageable, with the gold standard approach being a completion TME^52,53^. Minimally invasive surgical approaches, including laparoscopy or robotic surgery, are still feasible in this setting, as are sphincter-preserving operations. Combining surgical techniques, including transanal TME, may allow restorative surgery to be achieved in patients with very low rectal tumours who desire it. There is no additional morbidity conferred with delayed TME surgery compared to ‘immediate’ TME^66,67^. However, it is important to note that salvage surgery may be technically challenging due to the degree of radiotherapy-related fibrosis, which is reflected by the quality of the TME specimen being slightly worse in this cohort of patients^67^.

For a subgroup of patients, organ preservation, with LE for an LTR, is a potential option^68–70^. Data from the Dutch Watch and Wait Consortium suggest LE is associated with a 5-year overall organ preservation rate of 63%, 5-year colostomy-free survival of 68% and 5-year overall survival of 96%^68^. There were no differences in outcomes observed between patients with an early LTR (within 6 months of chemoradiation) and later LTR, that were then treated with LE. LE and organ preservation in the setting of LTR must be balanced against these long-term oncological outcomes. This approach is of most oncological value and benefit in earlier T stage and smaller tumours at baseline^71^.

### Functional outcomes of NOM *versus* TME

The comparable oncological outcomes of NOM and TME following neoadjuvant therapy place a greater emphasis on the functional outcomes of these different strategies^52,53^. One of the major proposed benefits of NOM is the avoidance of the surgical morbidity of major rectal resection and its longer-term sequelae on functional and quality-of-life outcomes. Pelvic radiotherapy is recognized to be an independent risk factor for surgical complications, namely anastomotic leak, and for poor postoperative function following surgery, including low anterior resection syndrome (LARS) and urinary and sexual dysfunction^72–75^. Although improvements in radiotherapy techniques may reduce toxicities, understanding the impact of pelvic radiotherapy alone within the context of NOM is essential to facilitate shared decision-making with patients, balancing oncological and functional outcomes, as well as overall quality of life.

Functional outcomes following treatment for rectal cancer focus on three key domains: bowel, urinary and sexual function. Neoadjuvant CRT followed by TME surgery is associated with significant post-operative dysfunction, with reported rates of LARS of 42–82.6%, urinary dysfunction of 20–77% and sexual dysfunction of 29–72%^76–79^. The degree and impact of dysfunction following NOM compared to neoadjuvant SCRT and surgery is reduced, with lower rates of LARS and urinary and sexual dysfunction reported across several cohort studies^80–84^. However, the relative preservation of function with an NOM approach is modest, and the functional impact remains significant^85^. Data from the Dutch registry report the incidence of major LARS at 2 years post-treatment as 25% following NOM. This is coupled with an incidence of moderate urinary dysfunction in 17.9% and severe erectile dysfunction in 30% of men at 24 months^86^. Female sexual function is also similarly impaired in patients undergoing NOM, with stable but moderate levels of dysfunction reported over a 2-year period^86^.

Health-related quality of life (HrQoL) is a complex construct, defined as a multidomain concept that represents the patient's general perception of the effect of illness and treatment on physical, psychological and social aspects of life^87^. Patients undergoing NOM have improved HrQoL scores across multiple domains, including physical, emotional, role and cognitive functions, compared to patients undergoing CRT and surgery^84^. This cohort of patients also reports overall better body image and fewer financial difficulties^84^. However, it is important to understand the trade-off for this cohort with regards to postoperative anxiety. When explored qualitatively, the emotional aspect of a NOM approach is significant, with anxiety, apprehension and fear of recurrence, future surgery and a potential stoma as predominant features^88^.

Overall, the evidence-base for HrQoL and functional outcomes following neoadjuvant chemoradiation and NOM is steadily growing, demonstrating a smaller magnitude of impact on functional outcomes and HrQoL. The emotional and psychological burden of NOM needs to be further evaluated and quantified, to ensure there is no adverse impact of anxiety and fear of recurrence on longer-term survivorship.

## Colon cancer

### Systemic chemotherapy

**Fig. 2:**
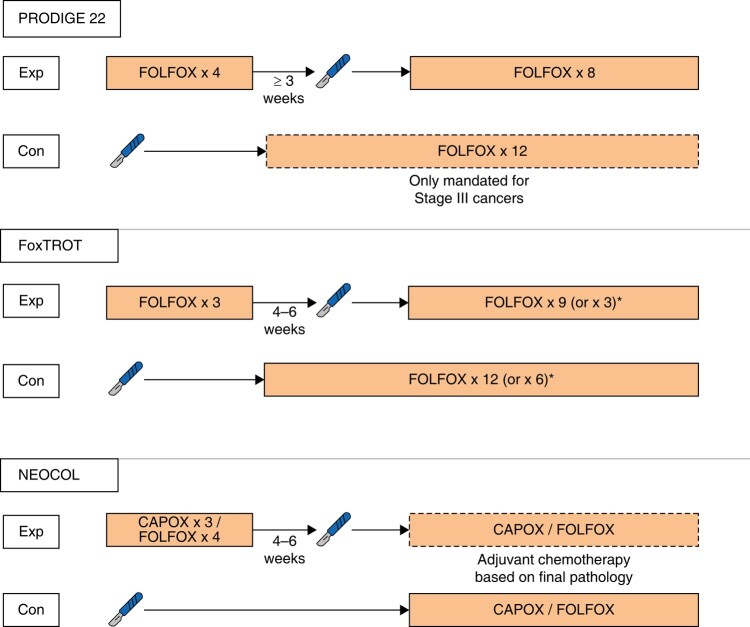
The treatment regimens used in the PRODIGE 22, FOxTROT and NEOCOL trials Exp, experimental arm; Con, control arm.

In contrast to rectal cancer, neoadjuvant therapy has a less-established role in the management of patients with colon cancer. Upfront surgical resection is currently the standard of care in colon cancer, with adjuvant chemotherapy recommended for patients with stage III disease and those with stage II disease with additional adverse features^89,90^. However, convincing arguments can be made for the need to intensify treatment in patients with high-risk colon cancers. Although there may have historically been more focus on surgical quality in patients with rectum cancer, recent studies suggest that the rates of R1 resection are just as high in patients with colon cancer and are associated with similar risks of both local and distant recurrence^91,92^. Prevention of systemic relapse presents a similar challenge in patients with colon cancer as it does in those with rectum cancer, developing in up to 25% of patients treated with adjuvant chemotherapy, with even higher rates seen in those with adverse risk features^93,94^. As such, arguments can be made for neoadjuvant treatment both as a means to reduce surgical complexity and the risks of systemic relapse in these patients. Given the benefits seen not just in rectal cancer but also in other gastrointestinal malignancies, several studies have investigated the potential benefit of neoadjuvant chemotherapy in patients with high-risk colon cancers, the designs of which are summarized in *Fig. 2* and the results summarized in *Table 3*. These have been limited to patients with locally advanced colon cancer, generally defined as those with cT3–4N0–2M0 disease.

**Table 3:** A summary of trials of neoadjuvant chemotherapy in patients with colon cancer

Study (year)	Inclusion criteria	*N*	Primary endpoint	Treatment arms	MPR rate (%)	R0 rate (%)	DFS (%)	OS (%)
PRODIGE 22 (2020)	cT3/4 or cN2	120	MPR	Perioperative FOLFOX	8	94	NS	NS
				Standard therapy	0	98	NS	NS
FOxTROT (2023)	cT3/4	1053	2-year residual/recurrent disease	Perioperative FOLFOX	8	94.9	16.9	85.0
				Standard therapy	0	89.7	21.5	82.2

DFS, disease-free survival; MPR, major pathological responses (defined as tumour regression grades 1 or 2); NS, not specified; OS, overall survival.

PRODIGE 22 was a randomized multicentre phase II trial treating 120 patients with either 12 cycles of adjuvant FOLFOX after upfront surgery or perioperative FOLFOX with four cycles delivered before surgery and eight cycles after^95^. The primary endpoint of the study was pathological response, and as such the study was not powered to detect any differences in survival. Compliance with perioperative chemotherapy was high, with >95% completing both the neoadjuvant and adjuvant courses, whereas only 73% in the standard arm started adjuvant therapy. Significantly more patients in the perioperative group had tumour regression, defined as tumour response grade 1–2 (44% *versus* 8%, *P* < 0.001). A trend towards downstaging was also seen, with fewer patients found to have stage III cancers on final pathological assessment in the interventional group. However, no corresponding differences in R0 rates were noted (94% *versus* 98%, *P* = 0.617). With longer-term follow-up, no statistically significant differences were noted in 3-year overall survival (90.4% *versus* 90.3%) or disease-free survival (73.0% *versus* 69.2%)^96^. Another important finding of this study was the high rate of clinical overstaging in the control arm, where 33% of patients were found to have low-risk stage II cancers on final pathological assessment.

The FOxTROT trial randomized 1053 patients to a similar FOLFOX regimen, although only three of the 12 cycles were given preoperatively in the perioperative chemotherapy arm^97^. The primary outcome of this study was residual or recurrent disease within 2 years of randomization. This endpoint was met, with a statistically lower recurrence rate observed in the perioperative chemotherapy group (16.9% *versus* 21.5%, *P* = 0.037). As in the PRODIGE 22 study, patients receiving perioperative chemotherapy had higher rates of pathological tumour regression (grades 1–2 in 8% *versus* 0%), and tumour downstaging (pT4 tumours in 21% *versus* 31%, *P* < 0.001). This time, however, a corresponding increase in R0 resections was also found compared to the control group (94% *versus* 89%, *P* < 0.001). Reassuringly, a trend towards reduction rather than an increase in perioperative complications was seen in the perioperative chemotherapy group. However, similar issues with clinical overstaging were noted, with 24% of patients in the control arm found to have pathological stage I or low-risk stage II cancers.

A different regimen was investigated in the NeoCol study, which has been published in abstract form^98^. Here, 248 patients were randomized to either upfront surgery or neoadjuvant chemotherapy with either three cycles of CAPOX or four cycles of FOLFOX. Adjuvant therapy was not given as standard but based on the final pathological staging. The primary endpoint was disease-free survival. As in the FOxTROT study, fewer perioperative complications were noted in the neoadjuvant group. However, no significant differences in 2-year disease-free survival or overall survival were observed.

Each of these three studies had an additional arm, investigating the potential benefit of targeted therapy with anti-epidermal growth factor receptor (EGFR) antibodies in patients with *RAS* wild-type cancers^95,97,98^. In patients with metastatic *RAS* wild-type colon cancers, the addition of anti-EGFR antibodies to FOLFOX is associated with improved survival when compared with FOLFOX alone^99^. However, no evidence of a corresponding benefit in the neoadjuvant setting has been noted. In the FOxTROT study, tumour regression rates and disease-free survival were no different in patients with *RAS* wild-type cancers allocated to FOLFOX with or without the addition of panitumumab^97^.

An additional important finding from the FOxTROT study is the impact of mismatch repair (MMR) status on the efficacy of neoadjuvant chemotherapy. Significantly more patients with proficient MMR (pMMR) cancers developed tumour regression when compared to those with deficient MMR (dMMR) cancers (23% *versus* 7%, *P* < 0.001). In keeping with this, although neoadjuvant treatment was associated with improved disease-free survival in patients with pMMR cancers, no apparent benefit was noted in patients with dMMR cancers. These differential effects according to MMR status mirror those seen in the adjuvant setting. Whereas adjuvant therapy is associated with a survival benefit in patients with pMMR colon cancers, no difference in survival was noted in those with dMMR cancers^100^. Furthermore, in patients with dMMR stage II cancers, adjuvant therapy appears to do more harm than good, being associated with reduced overall survival compared to surgery alone.

The precise role that neoadjuvant chemotherapy should play in patients with colon cancer remains uncertain. Neoadjuvant treatment is not mentioned in the current ESMO guidelines, which were published in 2020^89^. In contrast, the most recent NCCN guidelines, published in 2021, recommend the consideration of neoadjuvant chemotherapy in patients with ‘bulky nodal disease or clinical T4b’ cancers^90^. However, the limitations of current clinical staging, particularly regarding nodal stage, and concerns regarding the overtreatment of patients with low-risk cancers, are significant obstacles to the widespread adoption of this approach^101^. The development of more robust biomarkers for both disease stage and response to therapy may address these concerns.

### Intraperitoneal chemotherapy

An alternative strategy for colon cancer that has received recent interest is the intraoperative use of hyperthermic intraperitoneal chemotherapy (HIPEC). Although this may be considered more an immediate adjuvant rather than neoadjuvant therapy, these results are still worth discussing. The main aim of such a strategy is to reduce the risk of peritoneal metastases, which develop in up to 10% of all patients and are associated with reduced survival when compared to other metastatic sites^102,103^. Several studies have identified risk factors for the development of metachronous peritoneal metastases, such as locally advanced tumour (pT4 stage), tumour perforation, mucinous and signet ring cell histology, right-sided tumour location, nodal stage, and incomplete tumour resection (R1 and R2)^103–106^. HIPEC has several theoretical benefits such as a synergistic effect between hyperthermia and several chemotherapeutic agents, as well as high intraperitoneal doses that may kill free cancer cells and prevent their entrapment, in addition to reduced systemic toxicities compared with traditional chemotherapy^107^. However, the evidence of benefit from HIPEC as a prophylactic treatment for colorectal cancer is conflicting.

The recent Dutch COLOPEC trial assessed the potential benefit of adjuvant HIPEC. Patients with clinical or pathological T4N0–2M0-stage tumours or perforated colon cancer were randomized to adjuvant HIPEC followed by routine adjuvant systemic chemotherapy (experimental group) or to adjuvant systemic chemotherapy alone (control group)^108^. The adjuvant HIPEC protocol was a bidirectional chemotherapy containing intravenous 5-FU (400 mg/m^2^) and leucovorin (20 mg/m^2^) followed by intraperitoneal oxaliplatin (460 mg/m^2^) for a duration of 30 min at 42°C, delivered simultaneously or within 5–8 weeks after primary tumour resection. The primary endpoint was peritoneal metastasis-free survival at 18 months evaluated by diagnostic laparoscopy. A total of 204 patients were included. In the intention-to-treat analysis, there was no difference in peritoneal metastasis-free survival at 18 months (80.9% *versus* 76.2%; log-rank *P* = 0.28). The trial received several criticisms, especially regarding the choice of intraperitoneal chemotherapy as well as the timing of adjuvant HIPEC, and therefore a delay of adjuvant systemic chemotherapy. Furthermore, COLOPEC is now the third consecutive clinical trial that has failed to demonstrate efficacy of oxaliplatin-based HIPEC either as an adjuvant treatment or in patients with metastatic CRC, after ProphyloCHIP and PRODIGE 7^109,110^. Shared criticisms of these trials are that they all used a short duration of HIPEC of 30 min and used single-agent oxaliplatin, in mainly oxaliplatin pretreated patients, which were proven to be less effective in preclinical models as well as in patient-derived organoids^111,112^.

More positive results were seen in the Spanish multicentre clinical trial HIPECT4, where HIPEC was given as an intraoperative treatment at the time of initial surgical resection^113^. A total of 184 patients with locally advanced primary colon (cT4N0–2M0) were randomized to radical tumour resection plus HIPEC with mitomycin C (30 mg/m^2^; 60 min) compared to cytoreduction alone. The primary endpoint was 3-year locoregional control, defined as the absence of recurrence in the peritoneal cavity or tumour bed. The authors were able to demonstrate a significant improvement in the HIPEC group compared to the control group (97.6% *versus* 87.6%; *P* = 0.03). Reassuringly, no increase in morbidity rate was noted in the intervention arm or in the proportion of patients able to start adjuvant chemotherapy within 12 weeks of surgery. Despite the improvements in locoregional control, no corresponding improvements in disease-free survival or overall survival were noted. The contrasting findings of this trial highlight the importance of selecting the right HIPEC regime, both in terms of duration and the chemotherapeutic agents used. However, critics also highlight issues not only in terms of the statistical analyses of this trial but also with the potential overtreatment of patients found to have less-advanced cancer on final pathological staging. So, whereas the HIPECT4 trial has yielded more promising results than previous trials, the potential benefit of prophylactic HIPEC in patients with colon cancer is far from certain. Further work is needed to define what comprises the optimal HIPEC regime and identify which patients, if any, would benefit from its prophylactic use.

## Neoadjuvant immunotherapy for colon and rectal cancer

The results of recent early clinical trials of neoadjuvant immunotherapy in patients with colorectal cancer represent one of the most exciting breakthroughs in oncology in recent times^114–116^. Short courses of immune checkpoint inhibitors (ICIs) prior to surgical resection or as part of a NOM strategy have led to response rates far in excess of those previously seen with other neoadjuvant treatment modalities. However, the response to ICIs is not uniform and here, even more so than with neoadjuvant chemotherapy, MMR status appears to be a crucial biomarker for response.

ICIs work by enhancing the immune system's ability to detect and eliminate cancer cells. In order for them to have any effect, some degree of antitumour immune response must already be in process. Evasion of immune responses is increasingly recognized as a key step in cancer development and progression^117^. One of the most common mechanisms utilized by cancer cells is the manipulation of immune checkpoints, physiological pathways involved in the activation and regulation of immune responses. The most well-described of these pathways are the cytotoxic T-lymphocyte antigen 4 (CTLA-4) and programmed death 1 (PD-1) axes^118,119^. When activated, which they commonly are by cells within the tumour microenvironment, both pathways inhibit the activation of immune responses and therefore limit the immune system's ability to recognize and destroy cancer cells. ICIs block these pathways, thereby augmenting antitumour immune responses, and have led to dramatic alterations in the treatment and prognosis of many solid cancers^120–122^.

However, response to ICIs is known to vary both within and between cancer types, implying that some cancers are more readily recognized and attacked by the immune system than others. Several biomarkers for responses have been identified, which reflect either the immunogenicity of the cancer or the degree of antitumour responses already in process^123–126^. MMR status is one such biomarker and of particular relevance in colorectal cancer. Cancers with dMMR status are known to have a higher mutational burden than pMMR cancers. This in turn is predicted to result in a greater number of mutation-associated neoantigens^127,128^. These neoantigens are *de novo* proteins that are only expressed on the surface of cancer cells, which increase the likelihood of these cells being recognized as foreign by the immune system and then eliminated.

The value of MMR status as a biomarker has already been proven in metastatic colorectal cancer. ICIs first appeared to be ineffective in this setting when compared to other solid cancers, with no responses seen in a small, unselected cohort of seven patients with colorectal cancer^129^. However, a subsequent Phase II trial that stratified patients according to MMR status demonstrated marked activity in patients with dMMR colorectal cancer, with a response rate of 40%, whereas no responses were noted in patients with pMMR colorectal cancer^130^. Translational analyses from the same study found that response to therapy was associated with an expansion of T-cell clones reactive to neoantigens identified with the tumour^124^. Further studies have confirmed the activity of ICIs in dMMR metastatic colorectal cancer, with these agents now representing the first-line therapy for these patients^131,132^.

Encouraged by studies in other solid cancers, which demonstrated increased activity of ICIs in the neoadjuvant rather than adjuvant or metastatic settings^133–135^, several recent trials have investigated the potential of ICIs as a neoadjuvant treatment in patients with colon or rectal cancers. In the NICHE study, 40 patients with localized dMMR or pMMR colon cancers were treated with a single dose of the CTLA-4 inhibitor ipilimumab and two doses of the PD-1 inhibitor pembrolizumab followed by standard surgical resection performed within 6 weeks of inclusion^115^. Patients with pMMR colon cancers also received a COX-2 inhibitor, on the basis that this may increase response to ICI. The results were astonishing. All 20 of the patients with dMMR cancers responded to treatment, with major pathological responses (defined as ≤10% residual viable tumour) seen in 19 patients and a pCR seen in 12 patients (60%). Although not as impressive, increased activity when compared to the metastatic setting was also seen in patients with pMMR cancers, of whom 27% responded to therapy. The study treatment was also well tolerated. All patients completed therapy and underwent surgery as planned within 6 weeks of inclusion. Grade 3–4 treatment-related adverse events were seen in 15% of patients and anastomotic leaks occurred in four patients (10%).

The results of an expansion cohort (NICHE-2) including a total of 112 patients with localized dMMR colon cancers from the same research group have been published in abstract form^116^. This confirmed the high rates of response in these patients, with a major pathological response seen in 95% and a pCR in 67% of patients. Treatment-related adverse events were observed in three patients and three patients had delays of more than 6 weeks from inclusion to surgical resection. Although follow-up is limited, the responses to ICIs appear to be durable, with no local or distant recurrences observed in any of these patients to date.

Similarly impressive responses were observed in a small Phase II trial that included 12 patients with localized dMMR rectal cancer^114^. Patients with clinical stages II or III rectal cancer were eligible for inclusion and received nine cycles of the PD-1 inhibitor dostarlimab. In the original trial design, this was to be followed by CRT and then TME, with the option of NOM in patients who achieved cCR prior to surgery. Astonishingly, a cCR was noted in every single patient after the initial treatment with dostarlimab. Consequently, none of these patients received CRT or underwent surgery. Treatment was again well tolerated, with all 12 patients completing nine cycles and no treatment-related adverse events ≥grade 3 being observed.

Although the results from these early clinical trials still need to be validated in larger studies, immunotherapy will undoubtedly play a dominant role in the management of patients with dMMR colorectal cancers in the near future. However, whether that role will be to compliment or replace surgical intervention is yet to be determined. It seems obvious that ICIs should be considered as neoadjuvant therapies in patients with locally advanced dMMR colorectal cancers but do the costs, not only in socioeconomic terms but also with regard to potential toxicities, justify the use of these agents in patients with earlier disease stages? Should ICIs form the basis of NOM strategies in patients with dMMR colon cancers as well as rectal cancers? Perhaps, but that would require the ability to accurately discriminate between good responses and complete responses, which from anecdotal evidence appears to be a major challenge at present^136^. Although definitive answers to these questions are currently elusive, one thing is certain. With the advent of immunotherapy, the future treatment of patients with dMMR colorectal cancers will be markedly different from its current form.

## Future perspectives

Although significant advances in neoadjuvant therapy have been made in recent years, several major challenges remain. In patients with rectal cancer, one such challenge is the prediction and optimization of response to neoadjuvant treatment. The rates of pCR following SCRT or CRT for rectal cancer are around 20%, and although higher rates have been seen with TNT, the majority of patients do not achieve pCR. Improvement in pCR rates could not only improve long-term oncological outcomes but also increase the proportion of patients in whom NOM could be considered.

One potentially modifiable factor with the potential to influence response rates that has received much interest of late is the gut microbiome. *Fusobacterium nucleatum* has been particularly well studied in influencing neoadjuvant chemotherapy. Interest in *F. nucleatum* comes from the observations that tumour colonization by the microbe is associated with both poor survival and post-chemotherapy recurrence^137,138^. Yu *et al.* found that *F. nucleatum* mediated oxaliplatin and 5-fluorouracil chemoresistance via stimulation of autophagy-related proteins, suggesting that the worse outcome seen in colonized patients is potentially due to *F. nucleatum*-induced chemoresistance^138^. *F. nucleatum* has also been demonstrated to influence radiation therapy. Dong *et al.* found that in mouse models of colorectal cancer, oral *F. nucleatum* migrates to the tumour site and can elicit resistance to radiation^139^. Radiation resistance was associated with increased levels of both proliferative proteins (Ki-67, vascular endothelial growth factor) in cancer cell lines and a number of metastatic tumours in mouse models. Similarly, in a very recent 2023 report from 126 rectal cancer patients, *Bacteroides vulgatus* was found to attenuate the effect of chemotherapy and radiation^140^. The authors hypothesized that the underlying resistance mechanism was bacterial-promoted nucleotide synthesis. In addition to reports on individual strains, whole bacterial communities have been implicated in treatment response. Shiao *et al.* discovered that commensal bacteria were required for antitumour immune responses in breast and melanoma cancer lines^141^. Interestingly, targeting commensal fungi that enriched during antimicrobial treatment enhanced the response to radiation therapy, demonstrating that fungi similarly influence radiation toxicity. Future studies investigating other aspects of the gut microenvironment, such as the virome, will likely demonstrate further interaction that influences neoadjuvant treatments.

In addition to mechanistic studies, the components of the microbiome have also been investigated as potential biomarkers to predict tumour response to neoadjuvant therapy. Prospectively studying stool-associated microbiota in 84 rectal cancer patients, patients who had a significant response to chemoradiation had enrichment of butyrate-producing microbes, whereas non-responders demonstrated overrepresentation of Coriobacteriaceae and *Fusobacterium*^142^. White *et al.* similarly described bacterial changes that were associated with a response to therapy, but interestingly these alterations were dependent upon the age of the patient^143^. For example, in patients with early-onset rectal cancer defined as diagnosis <50 years of age, alterations of *F. nucleatum, Bacteroides dorei* and *Ruminococcus bromii* were all associated with tumour response. Alternatively, in patients older than 50 years, response to neoadjuvant therapy was only associated with *R. bromii*. Together, these studies indicate a complex host–microbiome crosstalk that underpins the development of rectal cancer and its response to treatment.

Patient selection represents a further major challenge, not only in terms of who should receive neoadjuvant treatment but also in the differentiation of good responses from complete responses in those patients who may be considered for NOM. Circulating tumour DNA (ctDNA) has recently emerged as a particularly robust and versatile biomarker in patients with colorectal cancer. Although data within this field is still maturing, there is already evidence to suggest that ctDNA may help address some of these issues. Dynamic changes in ctDNA during neoadjuvant therapy have an association with both pCR and risks of subsequent recurrence in rectal cancer. In a study of 159 patients with locally advanced rectal cancer, Tie *et al.* demonstrated that persistently positive ctDNA following CRT was associated with poorer recurrence-free survival following surgery (HR 6.6, 95% c.i. 2.6 to 17, *P* < 0.001)^144^. In a further study, Murahashi *et al*. found that a decrease in pre- to post-CRT ctDNA of ≥80% was an independent prognostic factor for pCR^145^. ctDNA has also demonstrated its value in detecting minimal residual disease in patients with stages II and III colon cancer^146,147^. These studies also demonstrated the utility of ctDNA in response assessment. In the study by Henriksen *et al.*, of the patients with positive ctDNA following surgery, only those who transitioned to being ctDNA negative following adjuvant chemotherapy did not develop recurrent disease^147^. A recent study by the same group also showed the versatility of ctDNA, where the specificity and sensitivity of these analyses can be adapted to specific clinical settings, with the aim of avoiding either overtreatment of low-risk patients or undertreatment of high-risk patients^148^. Although there are limited data regarding the use of ctDNA in patients receiving neoadjuvant chemotherapy for colon cancer, these data suggest that this biomarker could be used to help define complete responses following treatment.

## Summary

Neoadjuvant treatments for patients with colon or rectal cancers continue to evolve, increasing the complexity of decision-making for patients and clinicians alike. In patients with pMMR rectal cancer, TNT is likely to become the dominant neoadjuvant modality in the near future. NOM is increasingly preferred but demands frank discussions regarding the expected functional outcomes and the potential requirement for salvage surgery. Immunotherapy represents the most promising advance and is already dramatically altering the treatment paradigm for patients with dMMR colorectal cancers.

## Data Availability

Not relevant.
